# Establishment of Dental Pulp Cell Culture System for Analyzing Dentinogenesis in Mouse Incisors

**DOI:** 10.3390/dj13060270

**Published:** 2025-06-18

**Authors:** Yuka Kato, Insoon Chang, Satoshi Yokose

**Affiliations:** 1 Section of Endodontics, Division of Regenerative and Reconstructive Science, School of Dentistry, UCLA, Los Angeles, CA 90095, USA; ichang@dentistry.ucla.edu; 2 Division of Endodontics and Operative Dentistry, Department of Restorative and Biomaterials Sciences, Meikai University School of Dentistry, Sakado 350-0248, Japan; s-yokose@dent.meikai.ac.jp

**Keywords:** odontoblast, dentinogenesis, mouse dental pulp cells, primary culture, outgrowth

## Abstract

**Background**: The dentin–pulp complex plays a vital role in tooth health. Dentin forms the main body the tooth and continues to form throughout life to maintain homeostasis and provide protection against deleterious external stimuli. However, the detailed mechanism of dentin formation remains poorly understood, and there is a need for new regenerative therapies. This study therefore investigated whether primary dental pulp cells from mice could be used to establish a new culture system. **Methods**: Mouse mandibles were divided along the ramus to extract dental pulp tissue containing cervical loops. The extracted tissue was cultured in an incubator to promote cell out-growth and increase the number of cells available for experimentation. **Results**: Cultured cells formed mineralized nodules, confirmed by Alizarin red S staining. The expression levels of dentin sialo protein, bone gamma-carboxyglutamate protein, and type I collagen mRNAs in cultured dental pulp cells on day 15 were lower than those in intact mouse dental pulp tissue, and the expression of all mRNAs was confirmed through electrophoresis. **Conclusions**: This study established a primary culture system using dental pulp tissue extracted from mouse mandibular incisors. The results demonstrated that dental pulp cells can differentiate into odontoblast-like cells and form dentin-like mineralized nodules, thereby offering a useful system for studying dentin formation and odontoblast differentiation.

## 1. Introduction

Vital pulp therapy can contribute not only to the development of conservative dentistry but also the maintenance of oral health in a super-aging society. The evolution of vital pulp therapy could be supported and promoted through many kinds of basic studies of dentinogenesis in dental pulp cell biology. Suitable culture systems are particularly important for studies seeking to elucidate the mechanism of dentinogenesis. Numerous culture systems using cell lines and primary cells have been reported [1,2,3,4]. However, only a few studies using odontoblast cell lines established from dental pulp cells have been reported. There are only about four types of odontoblast cell lines, regardless of species, including MDPC-23 and MO6-G3. Of these, only two synthesize dentin-specific proteins [5]. This indicates that the establishment of odontoblast cell lines is very challenging, particularly cell lines that have characteristics similar to those of living odontoblasts. Furthermore, as Otsu et al. reported regarding the differentiation of induced pluripotent stem cells into dental mesenchymal cells, it will be difficult to obtain the cells [6]. Therefore, we established primary rat odontoblast-like cells [7]. However, since mice are smaller and easier to handle than rats, we aimed to establish a culture system for mice odontoblast-like cells. In the present study, we modified our previous method for establishing rat odontoblast-like cells to establish a culture system for odontoblasts in mice. Since dental pulp cells are composed of a heterogeneous population of undifferentiated mesenchymal cells, which are distributed from cell-dense areas to the center of the pulp and are associated with blood vessels, a system enabling the primary culture of dental pulp tissue could be a powerful tool for examining dentinogenesis. Furthermore, mesenchymal cells have the ability to differentiate into odontoblast-like cells [8]. In other words, since the cells present in dental pulp tissue are extremely diverse and not of a single type, primary culture is ideal for conducting research under conditions closest to those in the living body.

Primary culture systems are essentially divided into two categories depending on the method of cell isolation, either enzymatic digestion or out-growth. The former method has the advantage of enabling the collection of a large number of cells at once, but its main disadvantage is the requirement of a large amount of tissue. We previously reported a primary culture system using the enzymatic isolation of cells from rat incisor dental pulp tissues and demonstrated the system’s usefulness in analyses of dentinogenesis [7]. However, the dental pulp tissue of mice is much smaller than that of rats, and therefore, it is difficult to secure sufficient quantities for enzymatic treatment. For this reason, out-growth methods are considered beneficial for primary culture systems using mouse dental pulp tissue.

In this study, we developed a culture system using mouse dental pulp cells from the lower incisors with the out-growth method to facilitate examinations of the characteristics of odontoblasts and dentin formation. We also evaluated whether the differentiation and dentinogenesis processes of odontoblasts could be observed using these cultured cells.

## 2. Materials and Methods

### 2.1. Collection of Dental Pulp Tissue

All mice were cared for by the Division of Laboratory Animal Medicine (DLAM) at the University of California, Los Angeles (UCLA) following the DLAM protocols and US National Institute of Health guidelines. The mice used in this study met the approved standards of our institution’s animal care unit under the authority of UCLA, CSA (ARC-2024-078). Ten male and ten female 2- to 6-month-old mice were used for the collection of dental pulp cells. The method for extracting dental pulp tissue from the mandible of mice was developed with reference to the method reported by Yokose et al. [7]. Mice were handled so as to prevent pain and promptly used after sacrifice by inhalation anesthesia with isoflurane (Fluriso^TM^, Mwi Animal Health, Boise, ID, USA).

**Figure 1:**
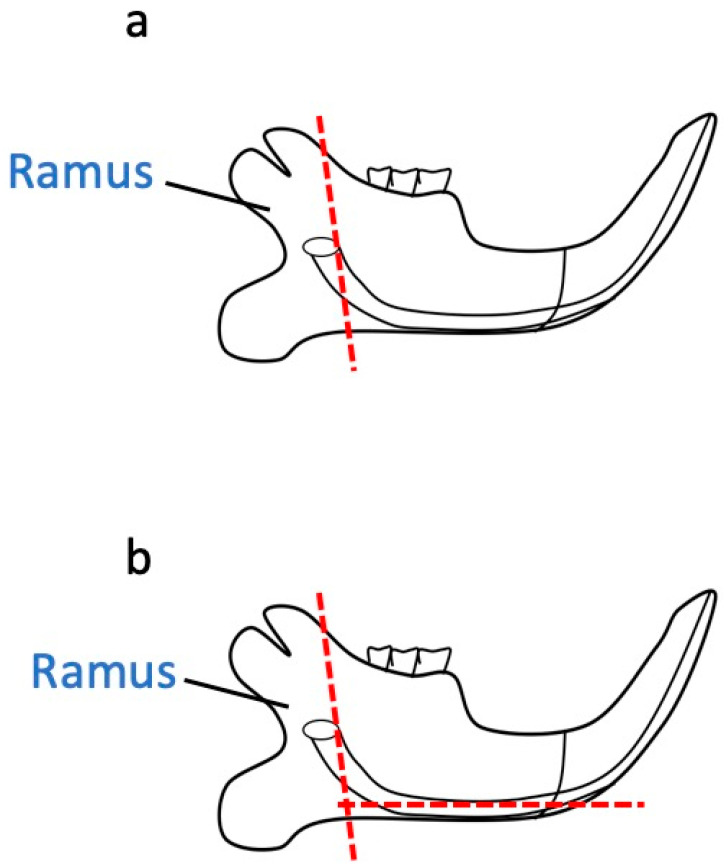
A schematic diagram of a mouse mandible. The red dashed line indicates the cutting position of the mandible for the collection of medullary tissue, including the cervical loop. We cut along the branches (**a**) and the angle (**b**) of the mandible.

First, the corners of the mouth on both sides were cut horizontally with scissors, and then the soft tissue between the lower lip and the mandible was bluntly detached, exposing the digastric muscle. With scissors, the mandible was divided in the sagittal direction in the center of the mandibular incisors, the soft tissue connected to the corpse was carefully removed, and the divided mandibles were removed one by one. Soft tissues, including the gums, attached to the removed mandible were carefully removed using gauze and tweezers. The mandibles of the mice were cut along the ramus and the angle of the jaw as indicated by the red line in Figure 1a,b, and pulp tissue containing the cervical loop was collected. After collection, the tissue was immediately placed in an incubator to allow out-growth and increase the number of cells for experimental use (Table 1).

### 2.2. Cell Culture

The tissues were inoculated into a 100 mm dish (Falcon Labware, Lincoln Park, NJ, USA) with 10% heat-inactivated fetal bovine serum (FBS; Gibco BRL, Gaithersburg, MD, USA) and 1% penicillin/streptomycin (Pen Strep, Gibco BRL, Gaithersburg, MD, USA) and incubated at 37 °C in the presence of 5% CO_2_.

Tissues and proliferated cells were washed with phosphate-buffered saline and collected using trypsin EDTA (Trypsin-EDTA Solution, Sigma-Aldrich, Rockvilie, MD, USA). The cells were centrifuged at 900 rpm for 5 min at 4 °C and seeded again in a 100 mm dish. After reaching a sub-confluent state, the cells were removed and cultured in a new 100 mm dish and incubated further. A sufficient number of cells in the second passage were then used for the experiment.

The collected cells were seeded in 12-well plates at a concentration of 5 × 10^4^ cells/cm^2^ and cultured in mineralized medium (MM), which consisted of 100 µmol/L ascorbic acid (Sigma-Aldrich), 2 mmol/L β-glycerophosphate (Sigma-Aldrich), and 10 nmol/L dexamethasone (Sigma-Aldrich) in α-MEM (Gibco) with 10% FBS and 1% penicillin/streptomycin [7,9]. The medium was changed every 2 days, and the cells were incubated up to day 15.

### 2.3. Alizarin Red S (ARS) Staining

On days 5, 10, and 15, the cells were rinsed with distilled water (DW) and fixed with 70% ethanol for 60 min at room temperature. ARS staining was performed (1% Alizarin Red S, Sigma-Aldrich), and then 10% CPC solution was prepared by adjusting the pH with NaH_2_PO_4_ and Na_2_HPO_4_, and the stained material was dissolved in the solution. That is, 800 μL of CPC solution was added to each well, and the wells were shaken to elute the ARS-stained material. After confirming that the material was completely dissolved, 100 μL was sampled from each well and placed in a 96-well plate. CPC solution was used as a blank, and the absorbance was measured at 562 nm. Data are reported as means and standard deviations (*n* = 9 in each group).

### 2.4. Real-Time Quantitative PCR (qRT-PCR)

Total RNA was isolated from cells cultured for 15 days in 12-well plates using TRIzol reagent (Invitrogen, Carlsbad, CA, USA). First-strand cDNA was synthesized from 600 ng of each total RNA sample using 1 μL of random hexamer (50 μmol/L, Invitrogen), and 1 μL of dNTPs (10 mmol/L, Promega, Madison, WI, USA), and then RNAse/DNAse-free water was added up to 10 μL; the samples were then incubated at 70 °C for 5 min and placed on ice for 1 min. Next, 2 μL of 10× M-MuLV buffer (NEB, Ipswich, MA, USA), 1 μL of RNase inhibitor (40 U/μL, NEB, Ipswich, MA, USA), 1 μL of MuLV reverse transcriptase (200 U/μL, NEB), and 6 μL of RNAse/DNAse-free water was added, and the samples were heated at 25 °C for 5 min, 42 °C for 1 h, and 70 °C for 20 min. qRT-PCR was carried out using a CFX96 real-time PCR system (Bio-Rad, Hercules, CA, USA) in 10 μL of solution containing cDNA (diluted 1:25), 0.2 μmol/L forward and reverse primers, dNTPs (10 mmol/L, Promega), and iQ SYBR Green Supermix (Bio-Rad) containing iTaqTM DNA Polymerase, MgCl_2_, and SYBR^®^ Green I. The PCR cycling conditions were as follows: 95 °C for 1 min (denaturation), followed by 40 cycles of 95 °C for 10 s, and 60 °C for 1 min (annealing/extension). qRT-PCR data were analyzed using CFX Manager Software, ver. 3.1 (Bio-Rad), and are reported as means and standard deviations (*n* = 9 in each group). The primers were purchased from Sigma-Aldrich, and their sequences are shown in Table 2.

The expression level of the target gene in each sample was normalized to that of *Gapdh* as a housekeeping gene. After amplification, a melting curve analysis of the PCR products was used to differentiate between specific and non-specific products. PCR products were confirmed by electrophoresis on 2% agarose gels using PAGE GelRed (Biotium, Fremont, CA, USA) for UV light transilluminator visualization. PureView100-bp DNA ladder (Azura Genomics, Raynham, MA, USA) was used as a marker.

## 3. Results

### 3.1. Microscopy

**Figure 2:**
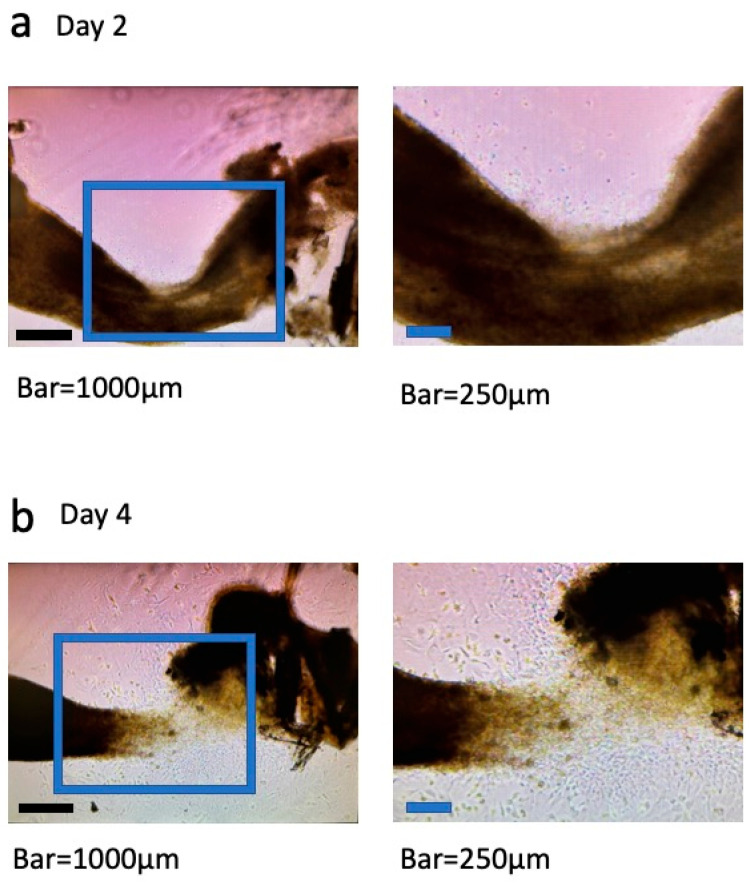
Photomicrographs taken on day 2 (**a**) and day 4 (**b**) after dental pulp tissue was placed in culture medium and allowed to out-grow. Bars = 1000 μm.

**Figure 3:**
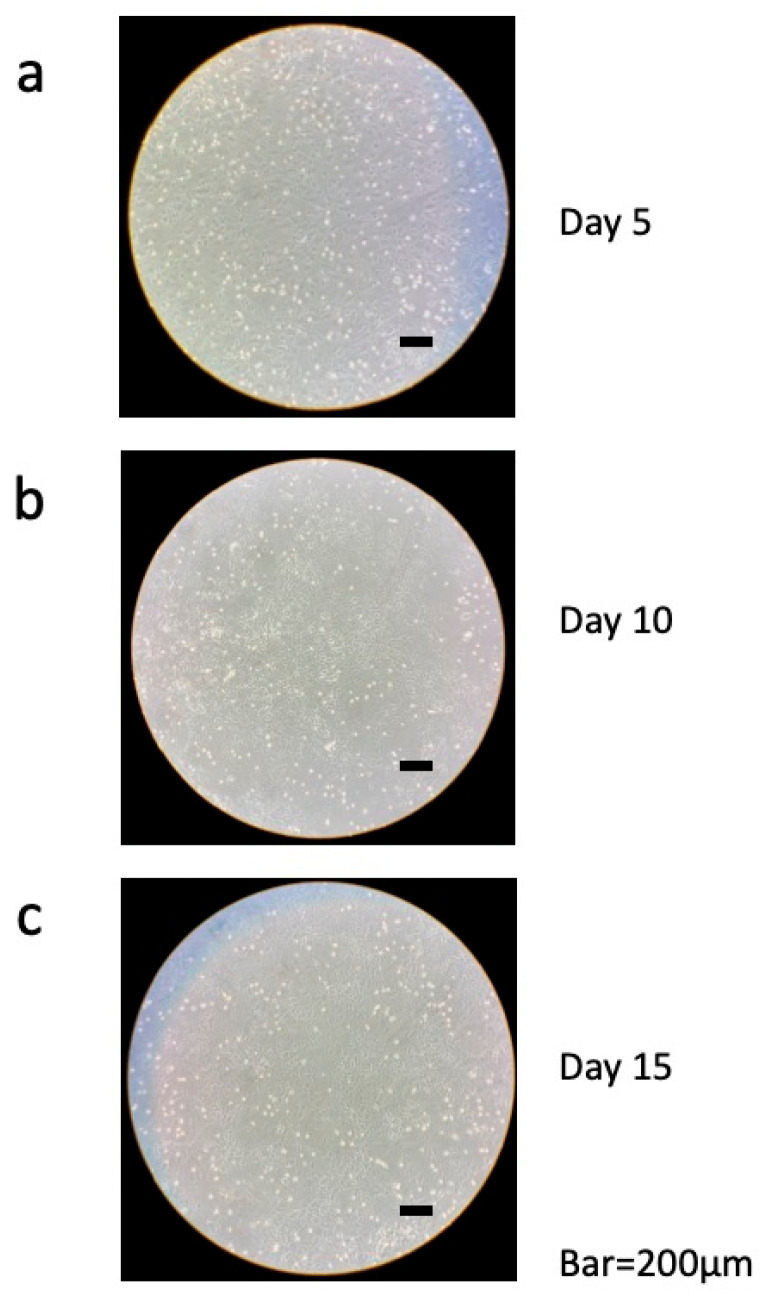
Photomicrographs taken on day 5 (**a**), day 10 (**b**), and day 15 (**c**) after dental pulp cells collected from out-growth were seeded into a 12-well plate. Bars = 200 μm.

The cultured dental pulp tissue was incubated in a dish, and cells grew out from the tissue. Figure 2 shows day 2 (a) and day 4 (b) of culture. On the left are low-magnification images and on the right are high-magnification images. On day 2, cells began to migrate slowly, and by day 4, we observed under the microscope that the tissue was breaking down and the cells were proliferating further. Cultured cells were incubated for 15 days, during which their state was periodically monitored under a microscope (Figure 3). On day 5 of culture, confluence was almost reached; gaps were observed between cells, and the shape of each cell could be clearly seen (Figure 3a). On day 10 of culture, the cells were lined up tightly and had reached full confluence. The cells were smaller in size than on day 5 of culture (Figure 3b). On day 15 of culture, as on day 10, the cells were arranged without gaps and appeared to have formed a network pattern (Figure 3c).

### 3.2. ARS Staining

**Figure 4:**
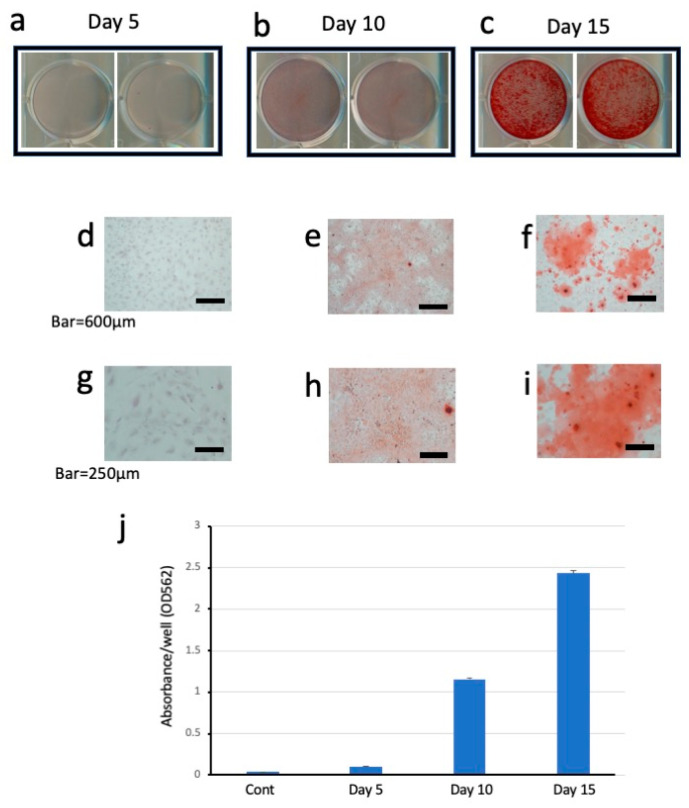
The staining of mineralized nodules formed in dental pulp cells cultured in MM on day 5 (**a**,**d**,**g**), day 10 (**b**,**e**,**h**), and day 15 (**c**,**f**,**i**). The top photographs show wells of 6-well plates stained on days 5, 10, and 15 of culture (**a**, **b**, and **c**, respectively). The middle photographs are low-magnification light microscopy images (**d**–**f**). The bottom photographs are high-magnification light microscopy images (**g**–**i**). Bars = 600 μm (**d**–**f**). Bars = 250 μm (**g**–**i**). On days 5, 10, and 15 of culture, the dye was extracted from the ARS-stained samples and the absorbance of the collected samples was measured at OD 562 nm (**j**).

Mineralized nodules of cultured dental pulp tissue were stained with ARS on days 5, 10, and 15 of culture (Figure 4). On day 5, no stained calcified nodules were observed (Figure 4a) either visually or under the microscope (Figure 4d,g). On day 10, some stained calcified nodules were observed (Figure 4b), and these stained cells were also detected under the microscope (Figure 4e,h). On day 15, the highest number of stained calcified nodules was observed (Figure 4c), and these stained mineralized nodules were also confirmed under the microscope (Figure 4f,i). The photographs on the right in Figure 4a–c show a single well of a 12-well plate. The photographs in Figure 4d–f show the histological features of the cells under a light microscope (bars = 600 μm). The photographs in Figure 4g–i show the histological features of the cells under a light microscope (bars = 250 μm). The graph in Figure 4j shows the data from days 5, 10, and 15 of culture. The dye was extracted from the ARS-stained samples, and the absorbance of the collected samples was measured at OD 562 nm.

### 3.3. Expression of Dentin Sialo Protein (Dsp), Bone Gamma-Carboxyglutamic Acid-Containing Protein (Bglap), and Type 1 Collagen (Col1a1) mRNAs in Dental Pulp Cells

**Figure 5:**
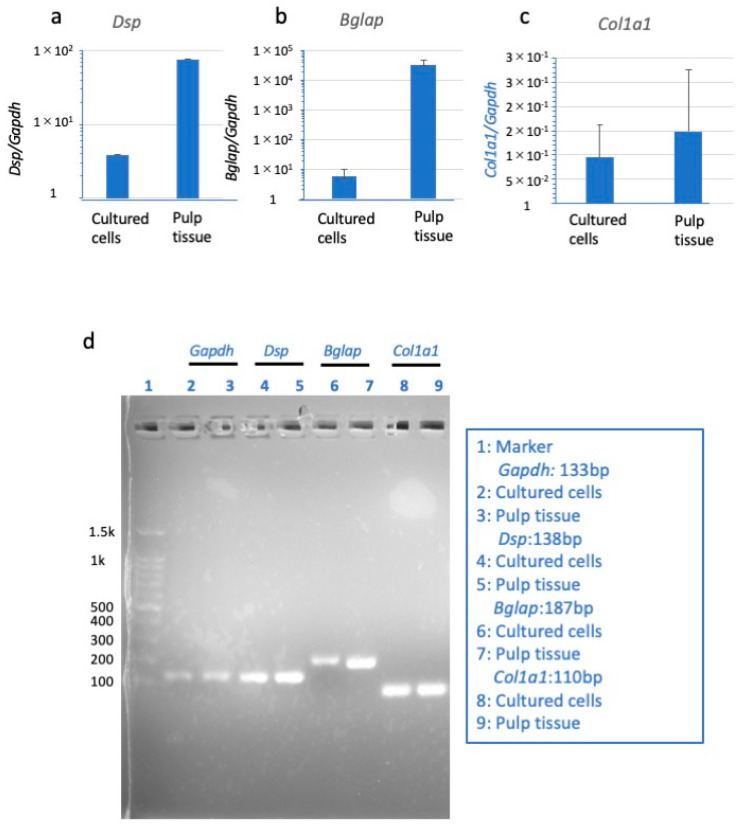
Expression of *Dsp* (**a**), *Bglap* (**b**), and *Col1a1* (**c**) mRNAs on day 15 in cultured dental pulp cells and cells of intact dental pulp. Target gene expression was normalized to that of *Gapdh*. The data are the means ± SD of triplicate cultures. PCR products obtained from qRT-PCR were electrophoresed to confirm product size (**d**). From the left, marker, (hereafter all are in the order of cultured cells and intact cells) *Gapdh*, *Dsp*, *Bglap*, and *Col1a1* are shown. PCR product size: *Dsp* = 137 bp, *Bglap* = 186 bp, *Col1a1* = 109.

qRT-PCR analysis was used to detect mRNAs of markers related to dentin formation in dental pulp cells and intact mouse dental pulp tissue. The expression levels of *Dsp* and *Bglap* mRNAs in cultured dental pulp cells on day 15 were lower than those in intact mouse dental pulp tissue; qRT-PCR analysis confirmed that all mRNAs were expressed (Figure 5a,b), which was further confirmed by electrophoresis (Figure 5d). These results confirmed that *Gapdh*, *Dsp*, *Bglap*, and *Col1a1* mRNAs, which are normally expressed during the formation of dentin-like mineralization nodules, were also expressed in cultured cells in this study (Figure 5).

## 4. Discussion

Mice and rats are widely used in biomedical research due to their genetic similarity to humans, small size, and short life cycle [10]. Rats have the advantage of being easy to dissect due to their larger body size relative to mice, and large amounts of sample can be collected from their excreta and organs. By contrast, the smaller size of mice compared with rats makes it difficult to extract tissues, but mice are easy to breed and exhibit high fertility and rapid generation turnover. Therefore, mice have been used as experimental animals for a long time [11] and for a variety of experimental purposes. The mouse genome has been completely sequenced, and considerable genetic information is available, making mice suitable for genetic experiments [12]. The availability of transgenic and knockout models in mice is also greater compared with rats [13,14]. Therefore, laboratory mice are often the model organism of choice in studies of human biology and disease, and we believe that the primary culture system for dental pulp cells described in this study will be very useful in dentinogenesis research in mice.

In order to increase the number of cells available for experimental procedures, we used dental pulp cells from the third passage. There was concern that repeated successive culture could result in the loss of odontoblast properties, but we confirmed that out-growth cells from mouse dental pulp tissues maintained their odontoblast properties. Notable formation of mineralized nodules stained with ARS was apparent by day 10 of culture and peaked on day 15. qRT-PCR analysis demonstrated that the cultured dental pulp cells expressed *Bglap*, *Dsp*, and *Col1a1* mRNAs on day 15 of culture. In addition, we reconfirmed that the PCR products obtained from cultured cells were identical to those obtained from the analysis of mouse dental pulp tissue, as shown by the electrophoretic analysis of the PCR products.

Dentin sialophosphoprotein (Dspp) is a non-collagenous extracellular matrix protein specifically expressed by odontoblasts [15,16]. Odontoblasts secrete Dspp as a phosphorylated parent protein that is post-translationally cleaved into two proteins, Dsp and dentin phosphoprotein [17,18,19,20,21]. Dsp is highly expressed in odontoblasts, but a small amount of expression has been observed in periodontal ligament cells, preameloblasts, and osteoblasts [22]. The results of our previous study using in situ hybridization in rat mandibles convinced us that *Dsp* expression was only observed in odontoblasts and no other cells [23]. Therefore, the cells expressing *Dsp* in the developed culture system could be considered to be odontoblast-like cells differentiated from dental pulp cells. It is possible that the dental pulp tissue isolated from the lower incisors of mice included the cervical loop containing enamel epithelium. It is well known that immature ameloblasts express *Dsp*, so the *Dsp* expression observed in the present study may have originated from ameloblasts in the cervical loop. However, Li et al. reported that ameloblasts are difficult to grow in culture beyond the second passage [24]. Moreover, Hermans et al. reported that primary ameloblasts fail to proliferate or survive in in vitro culture systems [25]. In the present study, we used the cells at the third passage and cultured the cells for 15 days. Therefore, it is extremely unlikely that *Dsp* was expressed from ameloblasts in the culture system.

Bglap is a Gla-protein related to matrix mineralization in dentin [26,27]. Research has shown that *Bglap* mRNA is expressed in fully mature odontoblasts at a high level during dentinogenesis [28]. Both Dspp and Bglap are secreted by odontoblasts, highly phosphorylated, and involved in dentin formation and mineralization. Dsp, Bglap, and Col1a1 are thought to play important roles in the formation of dentin. However, Bglap is expressed by both osteoblasts and odontoblasts and is closely related to matrix mineralization. In this study, it is considered that primary cells from the dental pulp tissue of mice lower incisors contained cells from several kinds of tissues, including osteoblasts. As a small amount of *Bglap* might be expressed by intermingling osteoblasts, we examined *Dsp* expression that was only expressed by odontoblasts. Furthermore, the expression patterns of *Bglap* and *Dsp* were the same and reflected mineralization. This suggested that the majority of *Bglap* was expressed from odontoblast-like cells. Our results indicate that mineralized nodules are not simply inorganic masses but contain organic matrix material that serves as an indicator of dentin formation.

## 5. Conclusions

Exploiting the out-growth potential of cells extracted from dental pulp tissue of mouse mandibular incisors, we established a primary culture system that modeled dentin formation. The results of this study suggest that our culture system is useful for analyses of the dentinogenesis process and could therefore contribute to the development of drugs and materials for vital pulp therapies.

## Figures and Tables

**Table 1:** Procedure for extracting mouse dental pulp cells.

1. The mandible of the mice were divided in the sagittal plane at the center of the incisors and removed in two pieces.
2. Soft tissues such as gingival tissue attached to the mandible were carefully removed using forceps, tweezers, and gauze.
3. They were then placed in 70% ethanol for sterilization and washed with PBS.
4. We made a sagittal cut with scissors along the mandible ramus, carefully widened the cut surface using a dental spoon excavator, and collected the pulp tissue, including the cervical loop, with forceps. The pulp tissue was carefully extracted in one piece as much as possible
5. The collected dental pulp tissues were cultured in an incubator (under 37 °C, 5% CO_2_) to allow out-growth with alpha MEM including 15% FBS and 1% penicillin/streptomycin.
6. The proliferated cells were collected using trypsin EDTA
7. The obtained cells were strained through a cell strainer and then used for all experiments.

**Table 2:** Sequences of primers used for qRT-PCR.

Primer	Forward (5′-3′)	Reverse (5′-3′)
*Gapdh*	ACAACTTTGGCATTGTGGAA	GATGCAGGGATGATGTTCTG
*Bglap*	CTGACCTCACAGATGCCAAGC	TGGTCTGATAGCTCGTCACAAG
*Dsp*	TACACCTCAGGGCTGGAAACTC	GTAGTCTCCAGACCTCGTAAGC
*Col1a1*	TAGGCCATTGTGTATGCAGC	ACATGTTCAGCTTTGTGGACC

## Data Availability

The original contributions presented in this study are included in the article. Further inquiries can be directed to the corresponding author.
